# Lake sedimentary DNA accurately records 20^th^ Century introductions of exotic conifers in Scotland

**DOI:** 10.1111/nph.14199

**Published:** 2016-09-28

**Authors:** Per Sjögren, Mary E. Edwards, Ludovic Gielly, Catherine T. Langdon, Ian W. Croudace, Marie Kristine Føreid Merkel, Thierry Fonville, Inger Greve Alsos

**Affiliations:** ^1^ Tromsø University MuseumUiT – The Arctic University of NorwayLars Thøringsvei 10N‐9037TromsøNorway; ^2^ Department of Geography and EnvironmentUniversity of SouthamptonSouthamptonSO17 1BJUK; ^3^ Laboratoire d'Ecologie AlpineUniversité Grenoble AlpesF‐38000GrenobleFrance; ^4^ Laboratoire d'Ecologie AlpineCNRSF‐38000GrenobleFrance; ^5^ Ocean and Earth ScienceUniversity of SouthamptonNational Oceanography CentreSouthamptonSO14 3ZHUK

**Keywords:** environmental DNA (eDNA), lake sediments, metabarcoding, sedimentary DNA (sedDNA), vegetation change

## Abstract

- Sedimentary DNA (sedDNA) has recently emerged as a new proxy for reconstructing past vegetation, but its taphonomy, source area and representation biases need better assessment. We investigated how sedDNA in recent sediments of two small Scottish lakes reflects a major vegetation change, using well‐documented 20^th^ Century plantations of exotic conifers as an experimental system.
- We used next‐generation sequencing to barcode sedDNA retrieved from subrecent lake sediments. For comparison, pollen was analysed from the same samples.
- The sedDNA record contains 73 taxa (mainly genus or species), all but one of which are present in the study area. Pollen and sedDNA shared 35% of taxa, which partly reflects a difference in source area. More aquatic taxa were recorded in sedDNA, whereas taxa assumed to be of regional rather than local origin were recorded only as pollen.
- The chronology of the sediments and planting records are well aligned, and sedDNA of exotic conifers appears in high quantities with the establishment of plantations around the lakes. SedDNA recorded other changes in local vegetation that accompanied afforestation. There were no signs of DNA leaching in the sediments or DNA originating from pollen.

## Introduction

Sedimentary DNA (sedDNA) from lakes has potential as a tool for reconstructing past vegetation (Anderson‐Carpenter *et al*., 2011; Pedersen *et al*., 2015; Thomsen & Willerslev, 2015). Even though studies of sedDNA show promising results (e.g. Willerslev *et al*., 2003, 2007, 2014; Pansu *et al*., 2015; Alsos *et al*., 2016), to date there have been few investigations concerning important aspects of sedDNA taphonomy, such as (1) the source area of plant DNA, (2) whether quantitative relationships exist between vegetation components and sedDNA, (3) how presence and absence are best defined when small quantities of sedDNA are present, and (4) whether sedDNA exhibits vertical mobility in a sedimentary column. Thus, further studies are necessary to reveal the full potential (and possible pitfalls) of using sedDNA as a proxy for vegetation composition (see Pedersen *et al*., 2015; Thomsen & Willerslev, 2015; Barnes & Turner, 2016; Birks & Birks, 2016).

Previous comparisons show that sedDNA and plant macrofossil records are floristically more similar than those of sedDNA and pollen. As pollen includes a substantial regional component, a primarily local origin of sedDNA is indicated (Jørgensen *et al*., 2012; Parducci *et al*., 2013, 2015; Pedersen *et al*., 2013). For lakes, we hypothesize that terrestrial plant DNA, which can be within plant fragments or bound as molecules to clay or organic particles, could be derived from anywhere in a lake catchment, transported via streams, ground water or overland flow, or eroded directly from the shore (see Barnes & Turner, 2016). If pollen were a source of sedDNA, pollen transported over some distance could complicate the interpretation of local vegetation, as it can in palynological studies (see Sjögren *et al*., 2008). Although DNA can be extracted and amplified successfully from pollen (Parducci *et al*., 2005; Keller *et al*., 2015; Kraaijeveld *et al*., 2015; Bell *et al*., 2016), it is less clear if pollen actually contributes to the DNA recorded in lake sediments (see Birks *et al*., 2012; Parducci *et al*., 2012a,b). It is therefore important to address if, and to what degree, different sources contribute to the sedDNA record. Finally, given the small molecular size of extracellular DNA, there might also be a possibility of movement with or through sediment pore water. Sporadic downward movement of DNA has been recorded in terrestrial sediments (Haile *et al*., 2007; Andersen *et al*., 2012), but it remains unclear if a similar phenomenon also occurs in lake sediments.

Only a few studies have addressed quantitative questions. Yoccoz *et al*. (2012) demonstrated a relationship between proportions of aboveground plant biomass and DNA abundance in soil, which, although noisy, suggests that the numbers of DNA copies (i.e. read numbers) may contain quantitative information. A modest but positive correlation has been found between environmental fish DNA read numbers in lake water and fish biomass (Evans *et al*., 2016). When low concentrations of DNA and/or degraded DNA are expected, such as in ancient DNA samples, the tally of a taxon's occurrence in multiple PCR repeats is suggested as a better determinant of its presence or absence than total sequence read numbers (Ficetola *et al*., 2015).

In order to evaluate how sedDNA records vegetation changes we took advantage of a ‘natural’ experiment. During the 20^th^ Century, large areas of previously open heathland and rough grazing land in southern Scotland were planted with non‐native conifers such as *Picea* sp. (spruce) and *Larix* sp. (larch). The native *Pinus sylvestris* (Scots pine) also was planted, augmenting a presence otherwise largely confined to dwindling semi‐natural woodlands. Although it is conceivable that DNA from ancient pine forests that resides in soils and sediments could be re‐deposited in the lakes, DNA of the exotic taxa must be related to modern afforestation. The plantations are well documented by the Forestry Commission (Scotland), and it is possible to determine when and where they were established. A vertical sequence of sediment samples from a lake within a plantation should thus, hypothetically, provide a sedDNA record describing an abrupt and significant change from ‘conifer‐free’ open heathland‐pasture communities to DNA assemblages dominated by coniferous taxa. The experimental setting mimics common applications of palaeoecology, namely, to determine when tree species colonized an area or how vegetation changed with alterations in land‐use. We analysed sedDNA in two short sediment cores from two Scottish lakes situated within well‐documented conifer plantations. The sedDNA records were complemented with pollen data, which allowed a direct comparison with a standard, well‐studied palaeoecological proxy. We had the following aims:

1. Investigate how the major changes in catchment vegetation composition are reflected in the quantity and quality of the sedDNA record. *Larix, Picea* and *Pinus* are distinguishable in our DNA reference library, and thus the expectation is that their DNA will appear in the sediment record at the depth that corresponds to first planting within the lake catchments, or soon after.
2. Compare the sedDNA record with the pollen record. This allows us to examine differences in sedDNA taxonomic resolution, source area and source dominance in relation to pollen. The contribution of nonlocal pollen can be documented and checked against the sedDNA record.
3. Assess whether there is vertical movement of sedDNA within lake sediments. The appearance of exotic conifer DNA in the sediment should be abrupt and coincident with the planting horizon, as determined by the isotopically estimated sediment age. Downward leaching would thus lead to an earlier than expected appearance of the DNA.

## Materials and Methods

### Study sites and field sampling

**Figure 1:**
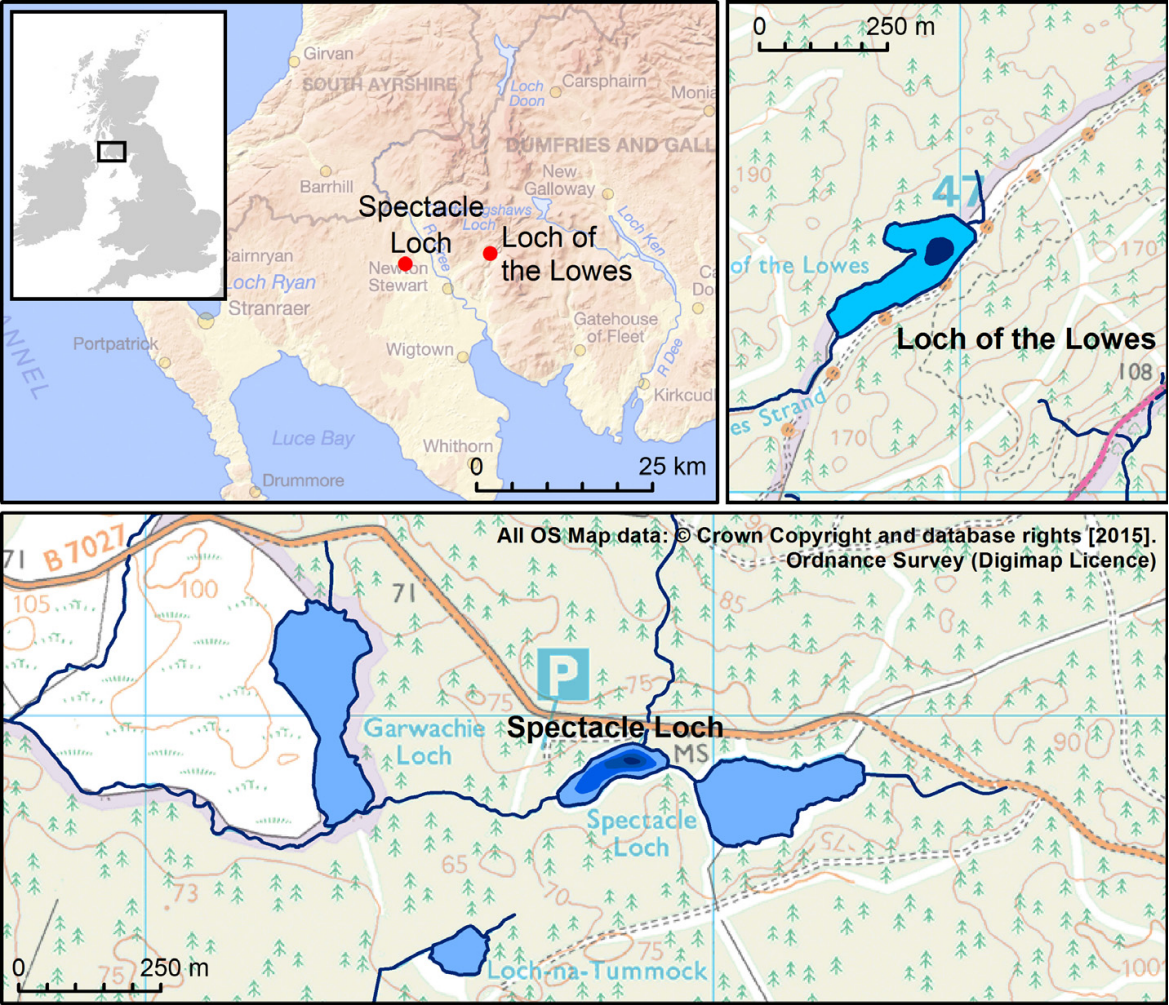
Overview and site maps. The bathymetric contours are approximate and delineate: 5‐m depth at Loch of the Lowes; and 3‐, 5‐ and 7‐m depths at Spectacle Loch. Cores were retrieved from the deepest part of each lake.

The study area lies in Galloway and Dumfries, vice‐county of Wigtownshire, southwest Scotland, a region that has undergone extensive afforestation in the 20^th^ Century (Fig. 1). We sampled two lakes within afforested catchments for short sediment cores: Loch of the Lowes (*c*. 3 ha, 55.004°N, −4.395°W, catchment *c*. 80 ha) and Spectacle Loch (*c*. 1.5 ha, 54.986°N, −4.579°W, catchment *c*. 230 ha). We used a Uwitec^™^ gravity surface sampler (60 mm diameter) from an inflatable dinghy. The lakes were sampled in the deepest part (see Fig. 1), at 5.5 m in Loch of the Lowes and 7.8 m in Spectacle Loch. Approximately 35 cm of sediment was recovered in both lakes. Immediately upon sampling, excess water was removed and the core top stabilized with Zorbitrol ^™^ gel. Cores were returned to the Palaeoenvironmental Laboratory at the University of Southampton within 2 d and stored at 4°C, then subsequently frozen and shipped to the Tromsø University Museum.

### Historical record

Based on historical Ordnance Survey (OS) maps (EDINA, 2015a,b) and forestry plantation maps (Forestry Commission Scotland, planting maps: Loch of the Lowes, 2012; Spectacle Loch, 2000) the main changes in vegetation around the lakes could be reconstructed back to the mid‐19^th^ Century. On the OS map of 1850, all land around Loch of the Lowes is marked as open (Supporting Information Fig. S1a), and maps from 1900 and 1910 show no change in vegetation. Planting of conifers, mostly *Pinus sylvestris* but also some *Picea sitchensis*, started in 1938. The bulk of this planting was in an adjacent catchment and only a small area was planted in the Loch of the Lowes catchment. *P. sylvestris* from this planting is still present today. By 1950, conifers had been planted widely southeast of the lake. Large areas on the northwest side were planted in 1962–1965, and in 1970 practically all land around the lake was afforested. In the 2000s, large areas around the lake were cut down and replanted, primarily with *P. sitchensis*. Trees stand close to the lake, but *Salix* spp., *Myrica gale* and *Calluna vulgaris* are prominent along the lake shore itself.

In 1840, Spectacle Loch was surrounded by rocky, heathy pastures, with marshland nearby (Fig. S1b). The nearest woodland according to the map was situated *c*. 1.5 km to the east. The situation was more or less the same in the 1950s. The area surrounding the lake (500 m radius) was planted with conifers between 1952 and 1962, with major plantings adjacent to the lake in 1960 and 1962. Afforestation continued with more planting in 1974–1976, and the entire area (> 1‐km radius) was covered with conifers by the late 1970s. In 2000, *Larix* forest and mixed *Picea–Pinus* forest dominated the surrounding vegetation (500 m radius), with *P. sitchensis, Pinus contorta* and *Larix kaempferi* all growing adjacent to the lake. Since then large areas north, east and south of the lake have been felled. In recent years (2008–2013) minor patches of native hardwoods (*Alnus*,* Salix* and *Betula*) were introduced, but not nearer to the lake than 300 m, and downstream of it. Around the shore there is abundant *Myrica* and *Calluna*, some *Salix* spp. and occasional trees of *Betula pubescens*.

### Sub‐sampling

Sub‐sampling, extraction and amplification set‐ups were performed in a laboratory dedicated for working with ancient DNA at Tromsø Museum. No PCR products have been present in this building. The cores were brought frozen to the laboratory and washed on the outside with chlorine solution. The frozen cores were sawed into 2‐cm‐thick slices; either *in situ* in the plastic tube or when the plastic was removed. All sampling equipment was washed in chlorine solution between the sampling of each slice. Sub‐sampling started from the bottom so if any extraneous material was moved, it was introduced upward, not downward. The sediment slices were put in zip‐lock bags and kept frozen. Subsequently the slices were allowed to thaw partially, then the outer material of each slice was removed with sterile scalpels (changed after each cut). Material for pollen analysis was taken from the edge of the ‘cleaned’ portion of the sample and the central sediment piece put into a tube for DNA extraction. Loss‐on‐ignition analysis and radiometric dating were performed on the remaining material.

### Core dating and age–depth assignments

Radiometric dating methods using ^137^Cs (half‐life of 30 yr) and ^210^Pb (half‐life of 22.4 yr) are well established (Croudace *et al*., 2012; Miller *et al*., 2014). ^210^Pb and ^137^Cs were determined at the National Oceanography Centre (Southampton) using Canberra well‐type HPGe gamma‐ray spectrometers (Canberra UK Ltd, Didcot, UK). Gamma ray spectra were acquired for 100 000 s for each sample (*c*. 2 cm resolution) and processed using Fitzpeaks gamma deconvolving software (JF Computing, Stanford in the Vale, UK). The anthropogenic radionuclide ^137^Cs shows three distinct datable features: the first appearance of ^137^Cs (~ 1954), the 1963 ‘bomb maximum increase’ and the 1986 Chernobyl event (e.g. Miller *et al*., 2014). ^210^Pb activity reaches zero at an age of *c*. 66 yr (*c*. 3 half‐lives of ^210^Pb), i.e. ~ 1950 ad (see Appleby & Oldfield, 1992; Croudace *et al*., 2012).

### Loss‐on‐ignition (LOI)

For LOI determination, Loch of the Lowes sediments from the same depths as the sedDNA samples were dried at 105°C overnight, placed in a furnace at 550°C for 4 h, and weighed after each treatment. For Spectacle Loch, we used freeze‐dried sediments representing 2‐cm portions of the sediment core, which were weighed, ignited at 550°C for 2 h and re‐weighed (see Heiri *et al*., 2001). The LOI values were calculated as ((dry weight – weight after burning)/dry weight) × 100.

### Pollen analysis

Pollen samples were prepared using the acetolysis method (Berglund & Ralska‐Jasiewczowa, 1986) and mounted in silicon oil for analysis. As our aim was to register the main changes in dominant taxa rather than to create a floristically detailed pollen diagram, an average of 180 grains were analysed per sample. Trends in DNA values and pollen percentages were compared for the more abundant terrestrial taxa (the planted conifers; *Pinus*,* Picea, Larix* and all taxa making up 5% or more of the sum of terrestrial pollen or DNA reads; *Quercus*,* Alnus*,* Betula*, Salicaceae, *Myrica*,* Calluna* and Poaceae). These taxa constitute on average 92.0% of the pollen assemblage in both Loch of the Lowes and Spectacle Loch.

### DNA analysis

Thirteen sediment samples from Loch of the Lowes, twelve sediment samples from Spectacle Loch, eight extraction negative controls and four PCR negative controls were processed. The sediment sample size was 5–8 g. DNA was extracted using the PowerMax soil DNA isolation kit (MO BIO Laboratories, Carlsbad, CA, USA). The manufacturer's instructions were followed, except that all centrifuge steps were done at 4800 ***g***, and, at step four, the samples were alternately placed in a water bath at 65°C and vortexed for a total of 30–60 min and 10 min, respectively. All samples were finally recovered in 3 ml of elution buffer.

Using a previously described protocol (Alsos *et al*., 2016), DNA was amplified and massively sequenced in parallel on a Illumina HiSeq 2500 platform, with the one change being that each sample (lake sediment as well as control) underwent six PCR repeats. Thus, the total number of repeats was 150 for the sediment samples (25 × 6) and 72 for the controls (12 × 6). All samples were pooled before sequencing. The short and variable P6 loop region of the chloroplast *trn*L (UAA) intron (Taberlet *et al*., 2007) was used as diagnostic marker, amplified with universal primers *‘g’* (5′‐GGGCAATCCTGAGCCAA‐3′) and *‘h’* (5′‐CCATTGAGTCTCTGCACCTATC‐3′). In order to segregate sequence reads bioinformatically and assign them to their relevant samples after high‐throughput sequencing, unique 8‐bp‐long tags (with at least five differences between tags) were added to the 5′ end of each primer (modified from Binladen *et al*., 2007 and Valentini *et al*., 2009).

Following the same analysis protocol (Alsos *et al*., 2016), next‐generation sequence data were aligned (*illuminapairedend*), filtered (*ngsfilter*) and trimmed (*obiuniq*,* obigrep* and *obiclean*) using the OBITools software package (Boyer *et al*., 2016; http://metabarcoding.org/obitools/doc/index.html). Resulting barcodes were then assigned to taxa using the *ecotag* program (Yoccoz *et al*., 2012) with both regional (Sønstebø *et al*., 2010; Willerslev *et al*., 2014) and global (EMBL release r117) reference libraries, as was done by Alsos *et al*. (2016). After data filtering, 11 171 750 reads of 17 145 unique sequences assigned to the 25 sediment samples were retained. A taxon was considered present in a repeat if it was a 100% match and was represented by 10 or more reads. For the full dataset, the limits for inclusion of taxa were as follows: two or more repeats in one sample, one or more repeats in two stratigraphically adjacent samples, or a total of four or more repeats anywhere in the core. These taxa were considered to have a strong enough DNA signal to justify further analysis.

Exotic taxa were checked for potential PCR errors and tentatively identified with BlastN 2.2.32+ (Zhang *et al*., 2000; Morgulis *et al*., 2008) to determine multiple/alternative taxon assignments (Table S1). Taxa assumed to be false positives based on their occurrences in negative controls were removed from further interpretation (see Table S2). All taxa identified in the DNA record after filtering were checked against the BSBI vice‐county records for Wigtownshire (VC74; http://www.botanicalkeys.co.uk/flora/vccc/index.html).

## Results

### Sedimentation, chronology and loss‐on‐ignition

The cores consisted of dark brown detritus gyttja with no visible changes in the sedimentation regime, as far as it was possible to determine given the frozen and partly covered state when sub‐sampled. The top parts of the cores from both lakes were observed in the field to be loose, flocculent sediment, and all samples became soupy after thawing.

**Figure 2:**
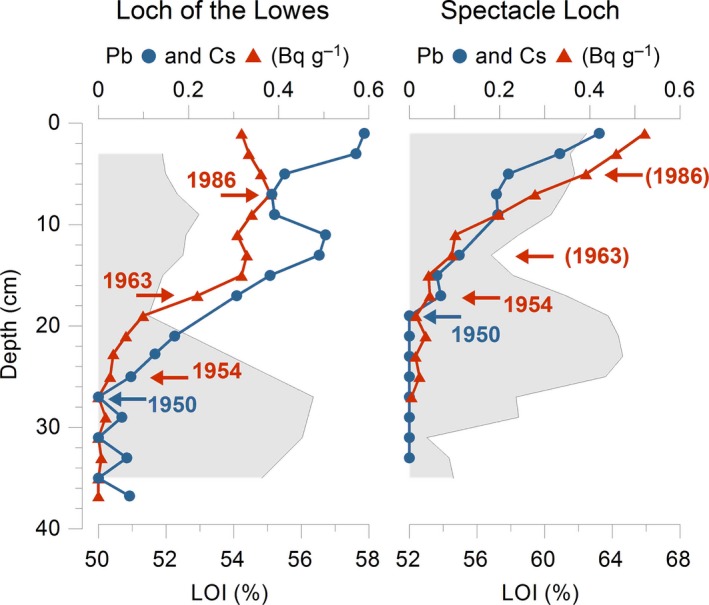
Radiometric sediment age estimations and loss‐on‐ignition (LOI). The middle minima in the LOI curves (shaded) are assumed to reflect increased erosion related to the main planting events. Dates are based on the first appearance of ^210^Pb activity (~ 1950) and the three ^137^Cs marker layers (the first appearance of ^137^Cs ~ 1954, the 1963 ‘bomb maximum’ increase and the 1986 Chernobyl event). Dating events within brackets are present but more subtle.

The ^210^Pb total and ^137^Cs profiles for each lake are fairly similar (Fig. 2). This implies that the erosion and transport of radioactively labelled soil particles can be correlated between the lakes, i.e. that the magnitude of disturbing activities and/or environmental change are similar. In Loch of the Lowes, the three ^137^Cs marker layers are seen in the data, whereas the Spectacle Loch profile the 1963 and 1986 events are present but more subtle; the 1963 and 1986 peaks are inferred from changes in the slope profiles (see Fig. 2).

**Figure 3:**
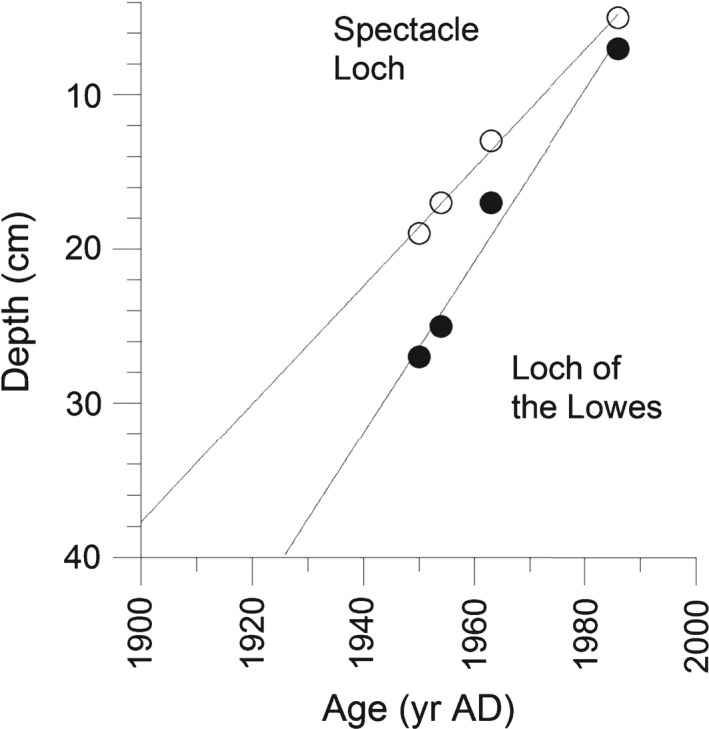
Age–depth models based on linear interpolation of ^210^Pb and ^137^Cs marker layers. The water–mud interphase is likely disturbed and not included in the models.

Age–depth models for the two lakes were constructed and show good linear correlations based on the four radiometric marker layers (Fig. 3). The sampling date (2012) for the sediment surface is not included because the sediment–water interface is likely to have been disturbed and/or lost during sampling. Sampling and measurement uncertainties are evaluated to be ± 1.0 cm for sample depth and ± 5% for ^137^Cs activities, based on gamma spectrometry counting statistics. The scale of these uncertainties does not have a significant impact on the age–depth model, and for the crucial period, 1940 to 1970, the ages are evaluated to be correct within a ± 5 yr range.

Upland afforestation typically involves disturbance of the soil surface; we anticipated that this might be recorded as an increase in minerogenic input as seen in the LOI values (Fig. 2). In Loch of the Lowes a decrease in LOI begins after 25 cm depth and ends at 19 cm, and in Spectacle Loch a decline begins after 20 cm depth and ends at 13 cm. According to the age–depth model the declines end at 1963 and 1965, respectively.

### Temporal changes in sedDNA

**Figure 4:**
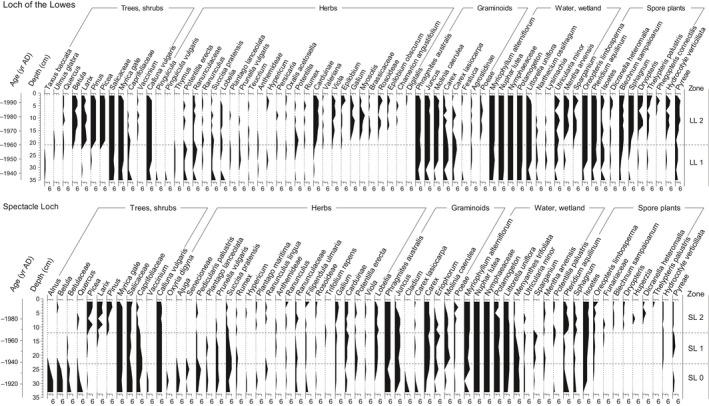
DNA repeat records (≥ 10 reads). Taxa with a minimum of two repeats in a single sample, a single repeat in two adjacent samples and/or ≥ four repeats in total are presented for each record. Taxa to the far right are aliens or common food plants (*Malus domesticus* included in Pyreae).

The results from the sedDNA analyses are presented as the number of DNA repeats vs depth, which provides information on presence–absence and a semi‐quantitative estimate of abundance (Fig. 4). Salicaceae, *Myrica gale*, and *Calluna vulgaris*, plus aquatic taxa (*Phragmites australis*,* Myriophyllum alterniflorum*,* Nuphar lutea*, Nymphaeaceae, *Potamogeton* and *Littorella uniflora*) are common throughout the sedDNA records of both lakes. Based on changes in the DNA values of the conifers and broadleaved trees, abundant taxa that show pronounced variations through the record, the Loch of the Lowes record was divided into two zones (LL1, LL2, visual inspection) and the Spectacle Loch record into three zones (SL0, SL1, SL2). Zones 1 and 2 are similar in both lakes and are interpreted together.

*Zone 0* (*c*. < 1910–1935) at Spectacle Loch pre‐dates the record from Loch of the Lowes. The DNA values for broadleaved trees (*Quercus*,* Alnus*,* Betula* and Salicaceae) indicate local presence of these taxa. *Calluna* is also well represented. The vegetation around the lake was likely an open heathland with scattered trees and/or small woods.

*Zone 1* (*c*. 1935–1960/65) is characterized by high DNA values of *Myrica*,* Calluna* and Poaceae and low values of all tree taxa, which suggests that open vegetation surrounded the lakes, probably *Calluna* heathland largely lacking local trees. This is in accordance with the historical maps depicting the local vegetation before plantation as heathy pasture.

*Zone 2* (*c*. 1960/65–2010) starts with an abrupt increase in sedDNA of *Pinus*,* Picea* and *Larix*, and declines in *Myrica*, Poaceae and *Calluna*, which indicate a rapid transition of the local vegetation from open heathland to conifer forest. This is in accordance with the planting of conifers as we know it from the historical records and the present vegetation. The continued presence of shrubs and grasses in the sedDNA likely reflects lake shore vegetation, small unplanted areas and/or relicts of heathland species in the undergrowth. In Loch of the Lowes sedDNA from *Quercus* and *Betula* appear, suggesting the local establishment of these taxa, likely facilitated by the cessation of grazing.

**Figure 5:**
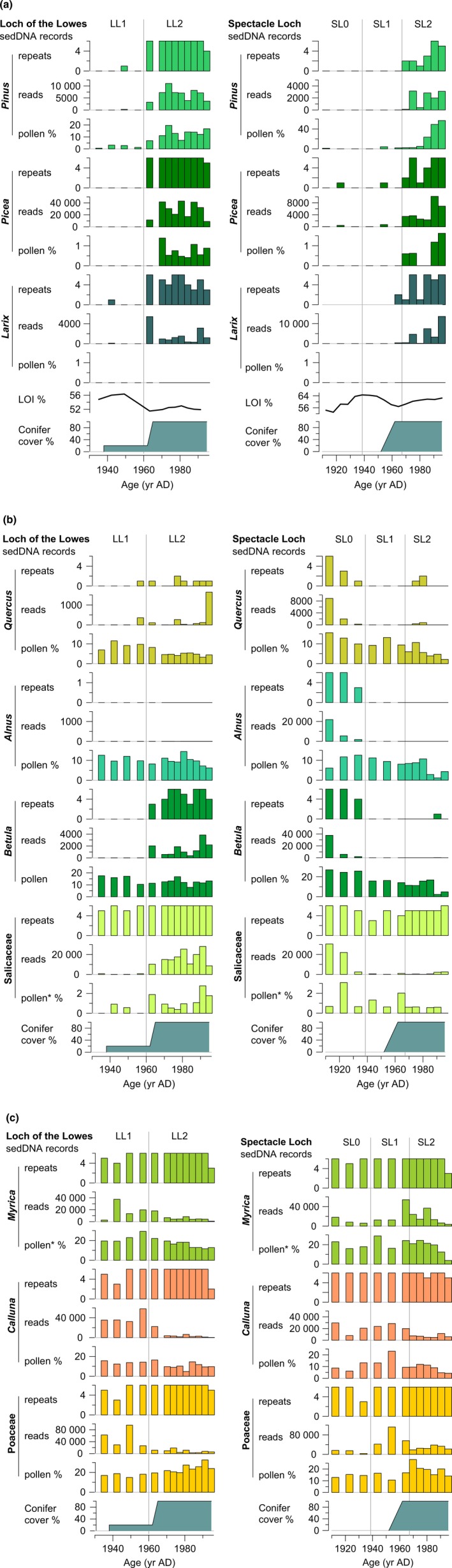
DNA and pollen results of selected taxa: (a) coniferous tree, (b) broadleaved trees, and (c) shrubs and grasses. Only planted conifers and terrestrial taxa with ≥ 5% of the pollen or DNA repeat records are presented. Pollen is presented as a percentage of the terrestrial pollen sum. LOI, loss‐on‐ignition; conifer cover %, percentage cover of conifer plantations within 500 m of the catchment in relation to modern planted areas as estimated from available historical maps. Note differences in scales on both *x*‐ and *y*‐axes. The youngest part of the age scale (> 1986) is extrapolated from the age‐depth model. *Salix and *Myrica*/*Corylus*‐type pollen in (b) and (c), respectively.

According to the radiometric dates, the boundary between zones 1 and 2, the main plantation event as detected in the sedDNA records (Figs 4, 5), occurred 1960 (~ ± 5 yr) in Loch of the Lowes and 1965 (~ ± 5 yr) in Spectacle Loch. Historical records show that early planting occurred at Loch of the Lowes in 1938, although the most prominent plantings occurred during 1962–1965. The decline in LOI dates to the early 1960s and thus corresponds with this main planting event (Fig. 5a). At Spectacle Loch, planting occurred between 1952 and 1962, with the main planting events in 1954 and 1960. The decline in LOI begins in the late 1950s, and conifer sedDNA values increase in the early 1960s. The dating of both lake records thus suggests that the time of the main planting aligns well with the appearance of exotic conifer sedDNA in the sediments, within the error of the methods.

Floristically, the DNA record is quite rich (73 unique native taxa), and most taxa are identified to genus or species. All taxa except *Hydrocotyle verticillata* are present in the vice‐county. Pyreae includes *Malus domesticus* (apple), and even if the wild species occurs in the region it is treated here as a potential food contaminant. Although it may be that some taxa present in vegetation surrounding the lake and as low reads/repeat numbers in the DNA data have been excluded, the fact that the DNA flora is ecologically appropriate indicates that the filtering procedures and thresholds for reads/repeats applied to the sequence data have effectively removed contaminants; the resultant taxa are likely to be true positives.

The record of herbaceous taxa at both lakes fits well with the change in land use during the 20^th^ Century. In zone LL1, a suite of taxa typical of moist moorland/rough grazing and/or the lake shore are present: *Pinguicula, Potentilla erecta, Ranunculus, Succisa pratensis, Plantago lanceolata*. These largely decline after the establishment of conifer plantations. A range of fern taxa, including *Blechnum, Dryopteris*,* Thelypteris* and *Phegopteris*, are far more common in the younger (plantation) zone, along with *Viola*,* Epilobium*,* Chamerion* and *Galium*. Similar changes occur in SL1, where species such as *Plantago lanceolata, Prunella vulgaris* and *Succisa* are more common pre‐planting and ferns appear post‐planting. Although many taxa occur in both zones, there is a clear switch in the dominant ones, reflecting a change in the field layer of the vegetation around the lake as grazing was reduced and the plantations developed.

The sedDNA read data (Fig. 5) show similar trajectories to the repeats for the conifer taxa and most of the broadleaved trees, but Salicaceae, *Myrica*,* Calluna* and Poaceae diverge. These are all relatively common taxa growing along the lake shores. On the one hand, their sedDNA repeat records indicate that they have been locally present throughout the period represented by the sediment columns. The record of sedDNA reads, on the other, shows major fluctuations, which may relate to quantitative variations in the abundance of the taxa. For example, *Calluna* shows an abrupt decline in sedDNA reads from zones 1 to 2, or when the open pasture‐heathland is replaced with conifer plantations.

When compared with historical maps, the numbers of reads and the historical abundance (area planted) of the different conifer taxa follow the same order at Loch of the Lowes: *Picea* is most common, then *Pinus*, then *Larix*. At Spectacle Loch, all three conifers have similar read values in the sedDNA, with exception of the uppermost samples, in which *Picea* and *Larix* are higher than *Pinus* (Fig. 5a). All three taxa have been planted adjacent to the lake, and, based on the maps, there is no clear difference in their abundance in the surrounding vegetation. Thus, the relative abundance of conifer sedDNA reads approximates the relative abundance (by area) of planted conifers at each site.

### SedDNA compared to the pollen signal

For full pollen diagrams and pollen concentrations of dominant taxa, see Figs S2–S5.

The pollen values of *Pinus* and *Picea* increase more or less simultaneously with the sedDNA or a little later; the lag is most clearly seen in the main rise of *Pinus* at Spectacle Loch (Fig. 5a). A delay in the pollen record compared with the planting event might be expected, as it takes time for the seedlings to reach maturity. A ‘tail’ of *Pinus* pollen (*c*. 2–4%) can be seen in both lakes, but this is likely a regional signal (but at Loch of the Lowes there was early *Pinus* planting). *Larix* is not detected by pollen analysis at these sites, which is likely an effect of its poor pollen dispersal capabilities and low pollen productivity (Sjögren *et al*., 2008, 2010).

Pollen values of *Quercus, Alnus, Betula* and *Salix* give a different picture of the surrounding vegetation development than does the sedDNA. In contrast to the sedDNA, pollen values show continuous presence and no sharp changes. This may reflect masking of local change by regional input. Notably, pollen from *Quercus, Alnus* and *Betula* are abundant in some zones where DNA is virtually absent (zone 1, and in Spectacle Loch also zone 2), which show that these relatively high levels of pollen do not provided a detectible DNA signal using the applied method.

*Myrica* and *Calluna* are well represented in both pollen and sedDNA. (The pollen type *Corylus/Myrica* likely includes a small proportion of *Corylus* pollen, however). Pollen and DNA reads follow roughly similar trends, but trends are different between sites (see Fig. 5c). Poaceae pollen values increase slightly from zones 1 to 2, but sedDNA reads decline. Poaceae includes many different species, and these may affect the pollen and sedDNA records differently. In particular, the emergent aquatic *Phragmites* is common and likely contributes high biomass and/or pollen load to the lake sediments, which could alter patterns of either or both proxies.

In the sedDNA dataset (150 repeats) and pollen dataset (~ 4500 terrestrial grains), we identified 97 individual native taxa (assuming that lower taxonomic units correspond to higher taxonomic units). Of these, 39 (40%) were unique to sedDNA, 24 (25%) unique as pollen and 34 (35%) recorded in both. Aquatic and spore taxa were better represented in sedDNA, with 14 (58%) unique to sedDNA, two (8%) unique to pollen and eight (33%) in common. It should be noted, however, that the number of identified taxa in both records depends on the particulars of the methods (e.g. number of DNA repeats per sample, representation of local flora in DNA reference library, number of repeats included to indicate presence, number of pollen grains counted, the pollen analyst's expertise and available time to identify rare pollen types, etc.). Thus, the above results are partly study‐specific.

## Discussion

Our goals were to assess how sedimentary DNA (sedDNA) records of plant taxa in lake sediments reflect known changes in vegetation cover and to compare the strengths and weaknesses of sedDNA in relation to pollen analysis. We were interested in the taxonomic clarity and temporal accuracy with which the sedDNA records afforestation, which indicate whether there has been downward leaching of sediments (or, conceivably, any laboratory contamination). The existence of interpretable quantitative trends and the degree of floristic detail available can both be assessed in relation to the pollen record. Finally, we can draw some conclusions about the source area for the sedDNA in these small lakes, based on observations of the catchments.

### The planting period: temporal precision

We know from historical sources that there was a major and relatively abrupt change in the vegetation surrounding the two lakes during the mid‐20^th^ Century. The sedDNA records major and abrupt change at the transition between zones 1 to 2: an increase in the conifers *Pinus*,* Picea* and *Larix*, and a decline in heathland taxa, especially *Calluna* (Figs 4, 5). At both sites, the sediment age estimations show that planting dates align well with the rise of the exotic conifer taxa in the DNA records. SedDNA thus works as a temporally and floristically accurate proxy for major changes in local vegetation at both lakes. Sporadic, low values of conifer DNA do occur before zone 2, but never exceed one repeat. It is not possible to distinguish such low levels of DNA detection from sequencing errors (Robasky *et al*., 2014), and in the present study we only regard conifer DNA values ≥ 2 repeats as proof for local presence of conifer trees (true positives).

The dating shows that these increases occurred *c*. 1960–1965, approximately at the time of the main planting events at the lakes. They are also associated with a slight loss‐on‐ignition (LOI) decline at both sites, which we interpret as reflecting soil disturbance due to mechanized activity, such as ripping of surface peat to improve drainage. This suggests that planting was largely complete before the DNA signal appeared in the lakes. At Loch of the Lowes, the relatively minor area planted in 1938 is recorded only as *Pinus* in a single sedDNA repeat, which is below what we considered certain detection level. Even if this planting was close to the lake it was not adjacent, and there are no major inlets on that side of the lake. Vegetation along the shores may thus have functioned to mask or filter the signal. It is likely that lake sedDNA primarily detects terrestrial vegetation that grow in the direct vicinity of the lake or inlet streams. The amount of biomass may also have played a role in when the sedDNA signal appears. Small seedlings may have grown for several years before their DNA reached the lake.

If DNA leaching occurred, we would expect DNA of exotic conifers before the time of plantation. The increases of conifer sedDNA between zones 1 and 2 are abrupt, and there is little in the way of ‘tails’ of lower values in preceding samples, suggesting that leaching is not a concern. In contrast to what has been found in two studies of terrestrial sediments (Haile *et al*., 2007; Andersen *et al*., 2012), our study does not indicate leaching of DNA in lake sediments. This is in accordance with other studies that also show that large organic molecules are immobilized in fine‐grained lake sediments (Smol, 2008).

### Floristic detail and plant abundance

The sedDNA provides quite a rich flora (73 taxa from 25 samples/150 repeats), and all but one taxon are recorded from the area in which the lakes are located. The absence of false positives is due to stringent filtering and application of thresholds to the data (see methods), a procedure broadly applied to environmental DNA records (Ficetola *et al*., 2015; Pedersen *et al*., 2015; Thomsen & Willerslev, 2015; Alsos *et al*., 2016). The sedDNA records several local plant communities. As expected, aquatic macrophytes are well represented, as they can be expected to contribute a large biomass to the lake sediments. In addition, aquatic plant material is less exposed to ultraviolet radiation and temperature fluctuation than terrestrial plant material before deposition in the lake sediments, which may improve the DNA quality (see Strickler *et al*., 2015). The main lake shore taxa such as *Salix* and *Myrica* are also abundant.

In addition to the changes seen in the tree taxa, the patterns for dominant herbaceous taxa in zones 1 and 2 tend to differ (Fig. 4), which shows that the sedDNA reflects changes in the field layer as well as in the dominant trees. The moist heath/rough pasture components of *Calluna* and a range of forbs show declines as the landscape becomes afforested, while fern abundance and species richness increases. This pattern is particularly clear at Spectacle Loch (see Fig. 4). Although we were not able to survey the vegetation of the two lake catchments exhaustively, the patterns of occurrence of the main taxa reflect well their current local presence or (near) absence, supporting our assumption that the sedDNA reflects the presence of local taxa, namely those within the hydrological catchment, especially aquatic and lake shore vegetation.

The variation in number of repeats can be related to the amount of DNA in the sediments, which, assuming all other factors being constant, in turn would be related to the abundance and proximity of the taxa in the surrounding vegetation (see Barnes & Turner, 2016). For most of the common taxa in our study, the number repeats reached maximum values, and were therefore noninformative about variations in abundance. For example, at Loch of the Lowes, *Calluna* and Poaceae show little change in repeat values through the record. However, both taxa show an abrupt decline in sedDNA reads from zones 1 to 2, when the open pasture‐heathland was replaced by conifer plantations (Fig. 5c). DNA read data could thus supplement DNA repeat data to detect shifts in abundance among common species.

### SedDNA compared with pollen

Our results show, as expected, that sedDNA and pollen data sense the surrounding vegetation differently. Pollen of anemophilous taxa, such as conifer and broadleaved trees, as well as most graminoids, has a large source area and potentially it could to a great extent represent regional vegetation (see Sjögren *et al*., 2008, 2010, 2015). DNA primarily represents local vegetation (near the lakes and within their hydrological catchments; Alsos *et al*., 2016). Pollen values are affected by species‐specific pollen productivity and dispersal properties (e.g. *Pinus* vs *Larix*), which are likely to affect DNA far less severely. In this test, using *Pinus*,* Picea* and *Larix*, we conclude that sedDNA gives a clearer signal of afforestation, particularly in the case of *Larix*. It also most likely gives a more realistic representation of the extent of broadleaved trees in the lake catchments in zones 1 and 2, particularly zone 2, when it is known that the catchments were afforested with conifers, yet there are still relatively high broadleaved tree pollen values. Our study strongly supports the idea that the DNA in lake sediments originates primarily from the hydrological catchment, as suggested previously. In particular, vegetation in or in direct proximity to the lake or inlet streams seem well represented.

In the present dataset there are several examples of high pollen abundance and no DNA presence for angiosperm taxa (e.g. *Quercus, Betula* and *Alnus* in zone 1; Fig. 5b). This suggests that sedDNA does not originate from pollen, at least for angiosperms. The reasons may be the relatively low biomass of pollen in sediment and/or the low copy numbers of cpDNA in pollen grains, as cpDNA is inherited maternally in most angiosperms (Nagata *et al*., 1999; Zhang *et al*., 2003; Ellis *et al*., 2008).

Our study does not allow us to say conclusively that DNA of conifers cannot be derived from pollen. In conifers, cpDNA is inherited paternally. However, previous studies indicate that even when pollen of gymnosperms is recorded, it may be absent in DNA from the same sample (Parducci *et al*., 2012b). In the present dataset, *Pinus* pollen do occur during the pre‐planting period with no clear correspondence in the sedDNA signal, although the pollen was present only in low abundances, so the evidence is not as clear as with the angiosperms. In the case of Loch of the Lowes we know that *Pinus* was planted at the lake during the zone 1 period, so neither can we dismiss the possibility that the weak DNA value that is present (one repeat) is related to biomass derived from vegetative remains. An obvious follow‐up experiment would be to assess DNA in a lake that has never had conifers in its catchment but is still close enough to a conifer stand to have high conifer pollen influx.

### Conclusions

The sedDNA records from Loch of the Lowes and Spectacle Loch accurately depict the major 20^th^ Century vegetation change – afforestation with conifers – known from historical data. The observed patterns are consistent with the sedDNA primarily reflecting local, within‐catchment vegetation, rather than the mix of local and regional vegetation portrayed by the pollen data. The level of floristic detail in the sedDNA is good and shows changes in minor as well as in dominant taxa. Aquatic taxa and taxa that dominate the biomass are especially well recorded, but forbs and cryptogams also are represented. The results of this study show that when carefully executed, sedDNA studies of lake sediments can provide reliable records, temporally and floristically, of local vegetation change. We recommend that future studies adopt the multiple‐repeat approach, which increases the probability of detecting rare species and provides good opportunities of semi‐quantification. For abundant taxa, the number of reads is more appropriate for semi‐quantification. Further calibration of source areas and spatial biases in lake sedDNA in a range of ecological settings is also desirable.

## Author contribution

M.E.E. and I.G.A. planned and designed the research; M.E.E. carried out the coring; L.G., M.K.F.M., P.S. and I.G.A. performed the DNA analysis; C.T.L. performed the pollen analysis; I.W.C. did the radiometric dating; T.F. compiled and interpreted the historical maps and planting patterns; PS analysed and presented the data; P.S. wrote the manuscript with input from M.E.E., L.G. and I.G.A.

## Conflict of interest

L.G. is one of the co‐inventors of patents related to g‐h primers and the subsequent use of the P6 loop of the chloroplast *trn*L (UAA) intron for plant identification using degraded template DNA. These patents only restrict commercial applications and have no impact on the use of this locus by academic researchers.

## Supporting information

Please note: Wiley Blackwell are not responsible for the content or functionality of any Supporting Information supplied by the authors. Any queries (other than missing material) should be directed to the *New Phytologist* Central Office.

**Fig. S1** Historical maps.
**Fig. S2** Pollen percentage diagram for Loch of the Lowes.
**Fig. S3** Pollen percentage diagram for Spectacle Loch.
**Fig. S4** Selected pollen and DNA data from Loch of the Lowes.
**Fig. S5** Selected pollen and DNA data from Spectacle Loch.
**Table S1** Re‐assignment of exotic taxa
**Table S2** Negative controls
