# Supplementary material for: Lake sedimentary DNA accurately records 20th Century introductions of exotic conifers in Scotland
Source: New Phytol. 2016 Sep 28;213(2):929–41. doi: 10.1111/nph.14199 (PMC5215665; doi:10.1111/nph.14199)
Supplement: Supplementary file 1 — Fig. S1 Historical maps. Fig. S2 Pollen percentage diagram for Loch of the Lowes. Fig. S3 Pollen percentage diagram for Spectacle Loch. Fig. S4 Selected pollen and DNA data from Loch of the Lowes. Fig. S5 Selected pollen and DNA data from Spectacle Loch. Table S1 Re‐assignment of exotic taxa Table S2 Negative controls [file NPH-213-929-s001.pdf]

New Phytologist

New Phytologist Supporting Information

Article title: Lake sedimentary DNA accurately records 20th century introductions of exotic conifers in Scotland

Authors: Per Sjögren, Mary E. Edwards, Ludovic Gielly, Catherine T. Langdon, Ian W. Croudace, Marie Kristine Føreid Merkel, Thierry Fonville and Inger Greve Alsos

Article acceptance date: 08 August 2016

The following Supporting Information is available for this article:

Fig. S1 Historical maps.

Fig. S2 Pollen percentage diagram for Loch of the Lowes.

Fig. S3 Pollen percentage diagram for Spectacle Loch.

Fig. S4 Selected pollen and DNA data from Loch of the Lowes.

Fig. S5 Selected pollen and DNA data from Spectacle Loch.

Table S1 Re-assignment of exotic taxa.

Table S2 Negative controls.

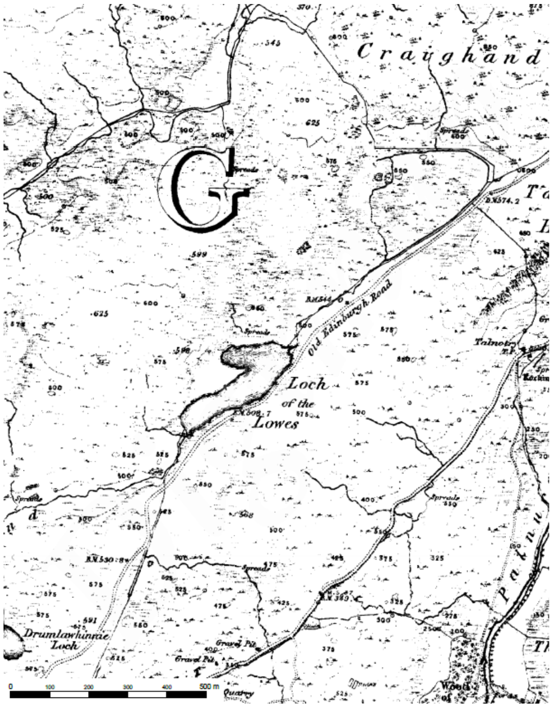

1850 – Heathy pasture

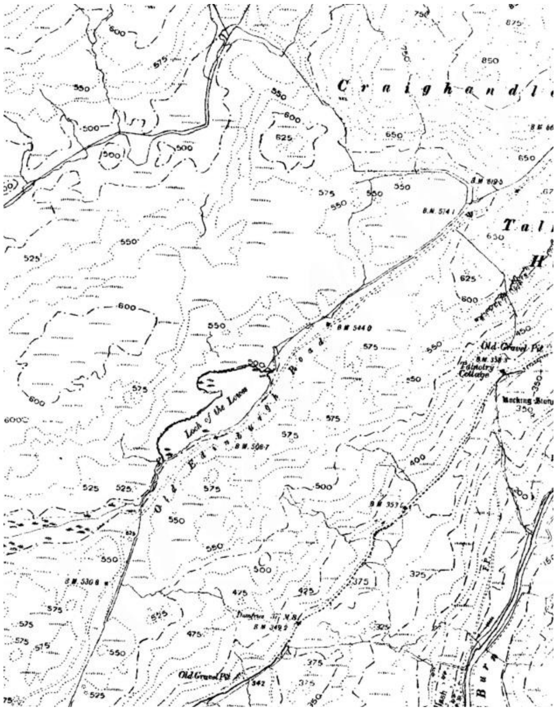

1910 – Heathy pasture

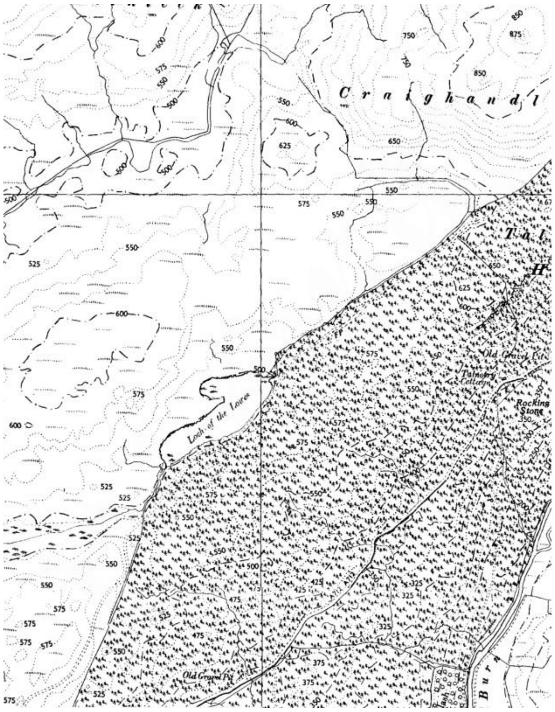

1950 – Heathy pasture, conifer forest

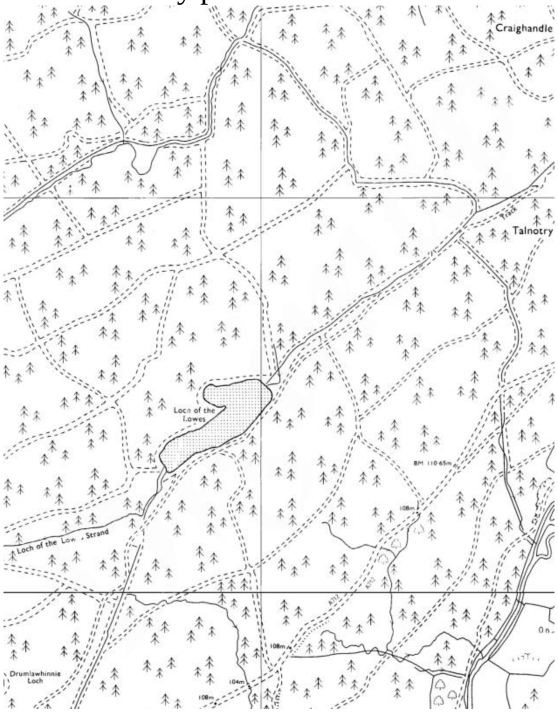

1970 – Conifer forest

Fig. S1a Historical maps of Loch of the Lowes. The scale bar in the top left image indicates 500 m.

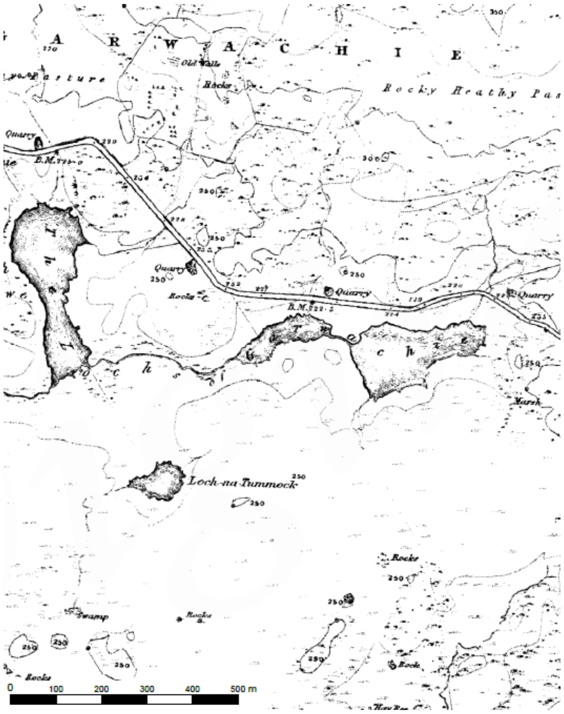

1840 – Heathy pasture

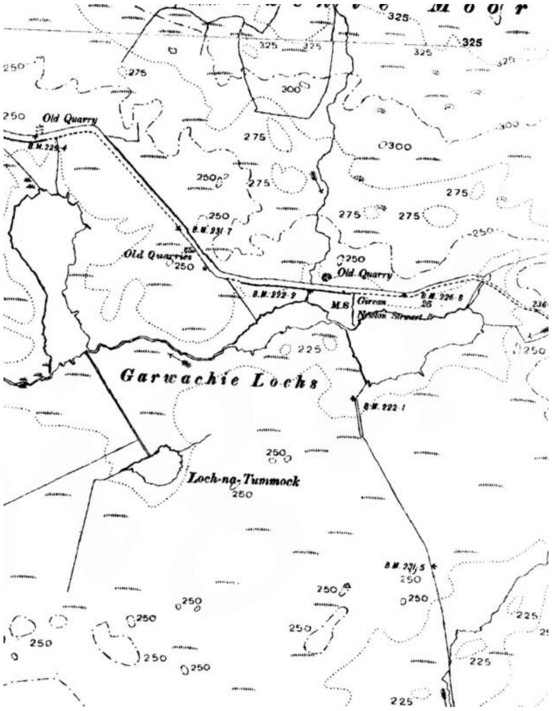

1900 – Heathy pasture

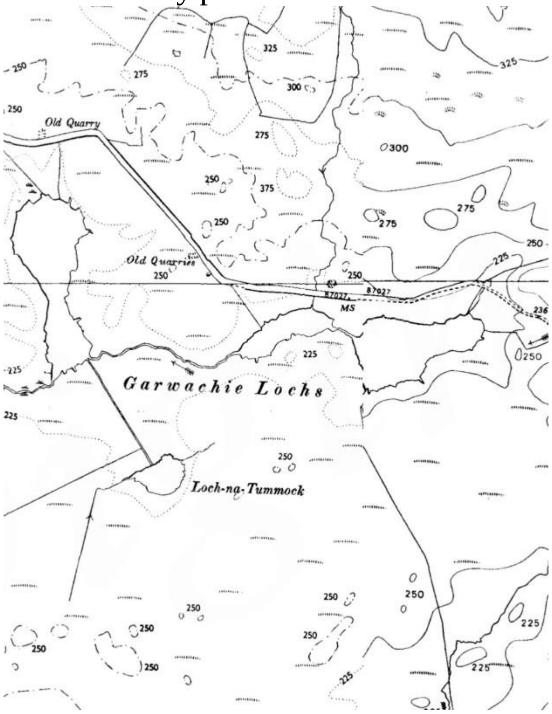

1950 – Heathy pasture

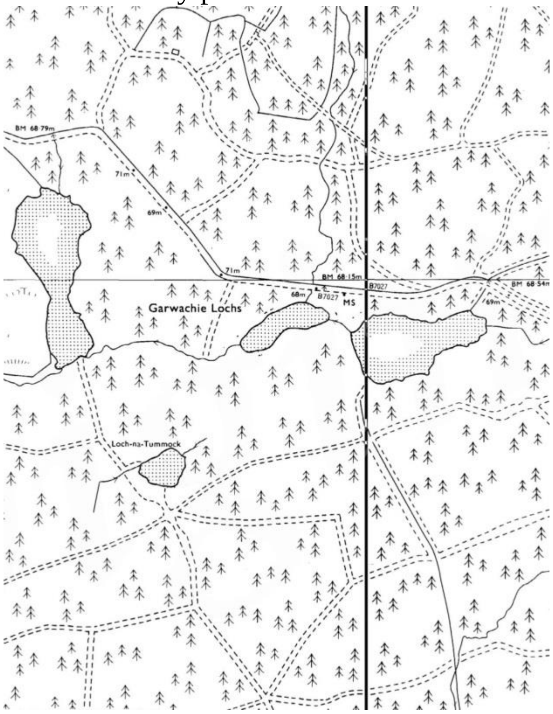

1970 – Conifer forest

Fig. S1b Historical maps of Spectacle Loch. The scale bar in the top left image indicates 500 m.

New Phytologist

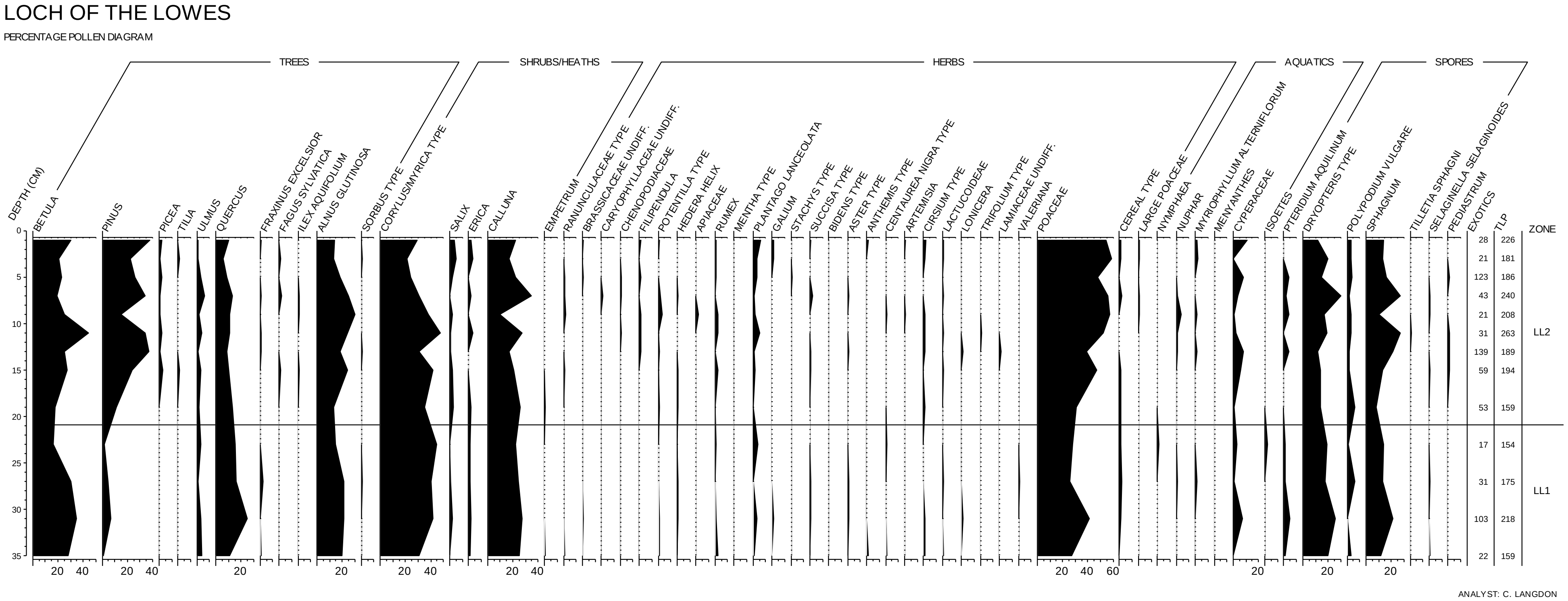

Fig. S2 Pollen percentage diagram for Loch of the Lowes. Percentage values are based on total land pollen (TLP).

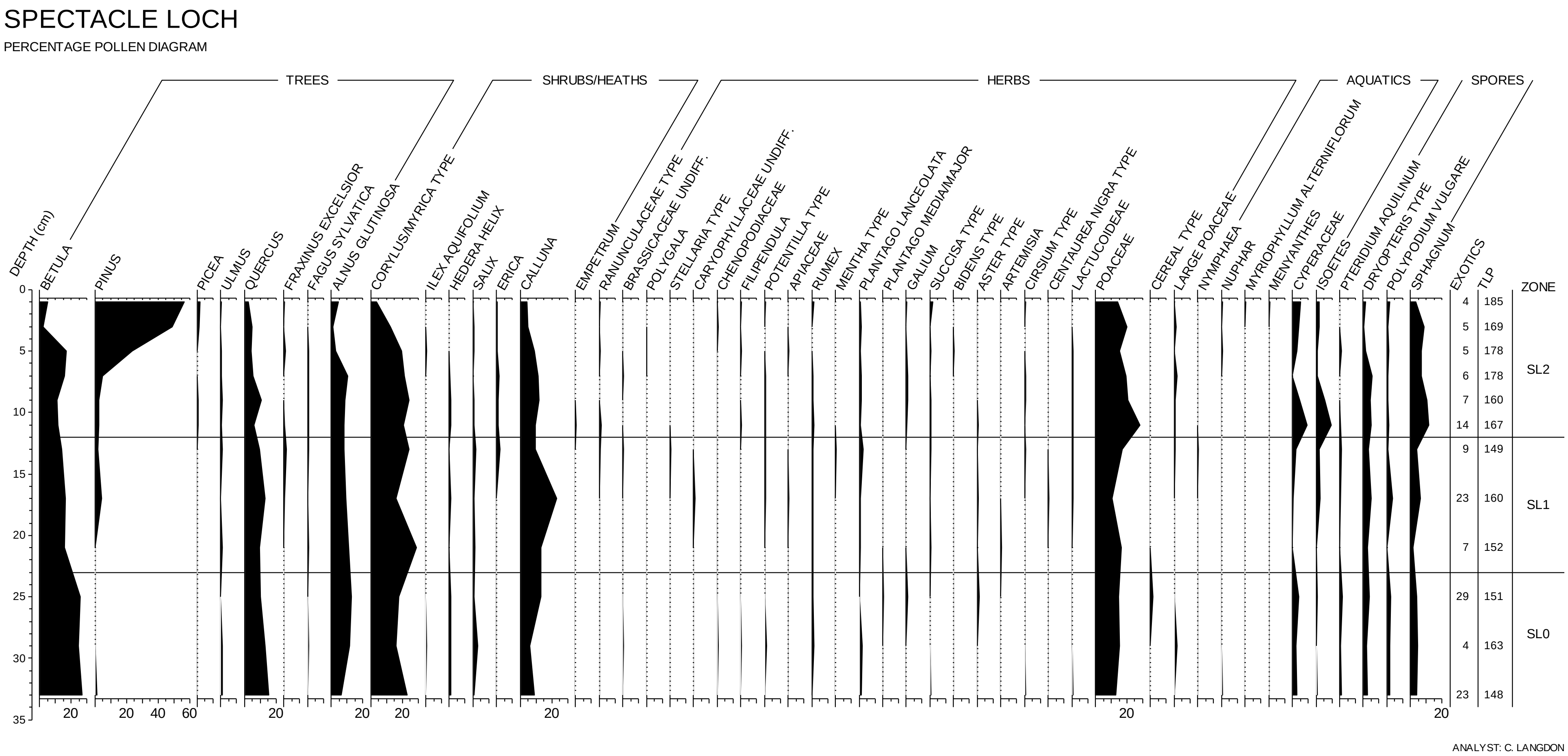

Fig. S3 Pollen percentage diagram for Spectacle Loch. Percentage values are based on total land pollen (TLP).

Loch of the Lowes

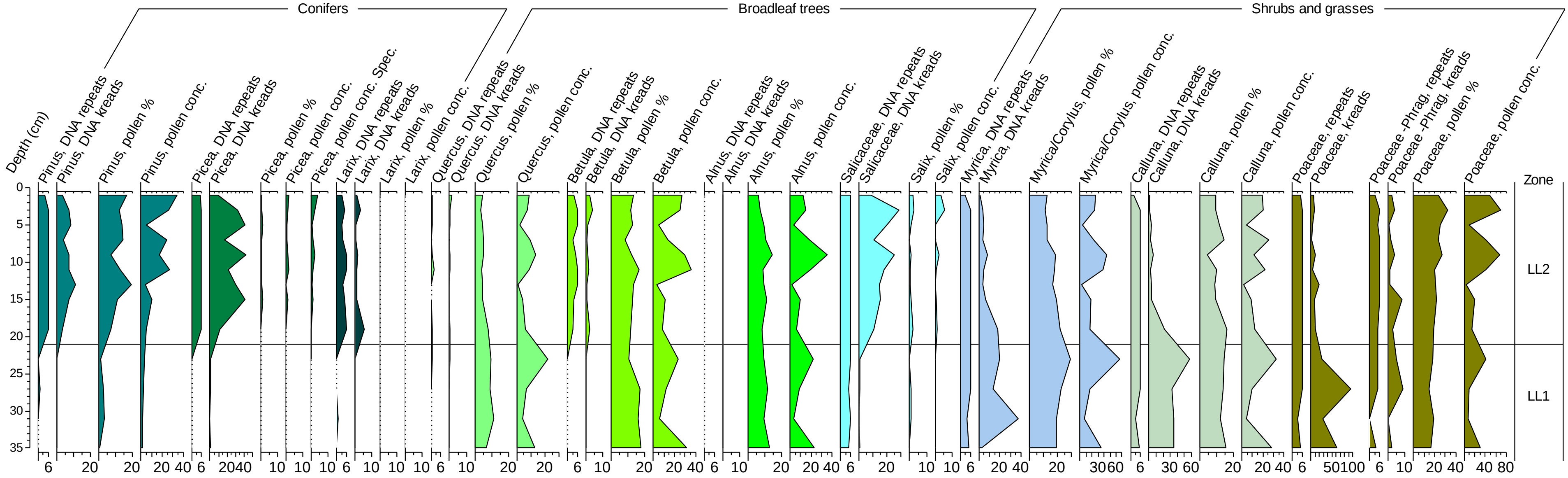

Fig. S4 Comparison of sedDNA repeats, sedDNA reads, pollen percentages and pollen concentrations from Loch of the Lowes, Scotland. DNA reads are given in 1000 of reads (sum of all six PCR repeats). Pollen percentage values (pollen %) are based on total land pollen. Pollen concentrations values (pollen conc.) are given as 1000 of pollen per cm^3. ″Picea pollen conc. spec″ is based on a higher count of exotics than done for the other samples. ″Poaceae -Phrag.″ is the remaining Poaceae DNA repeats and reads when *Phragmites australis* is removed.

New Phytologist

Spectacle Loch

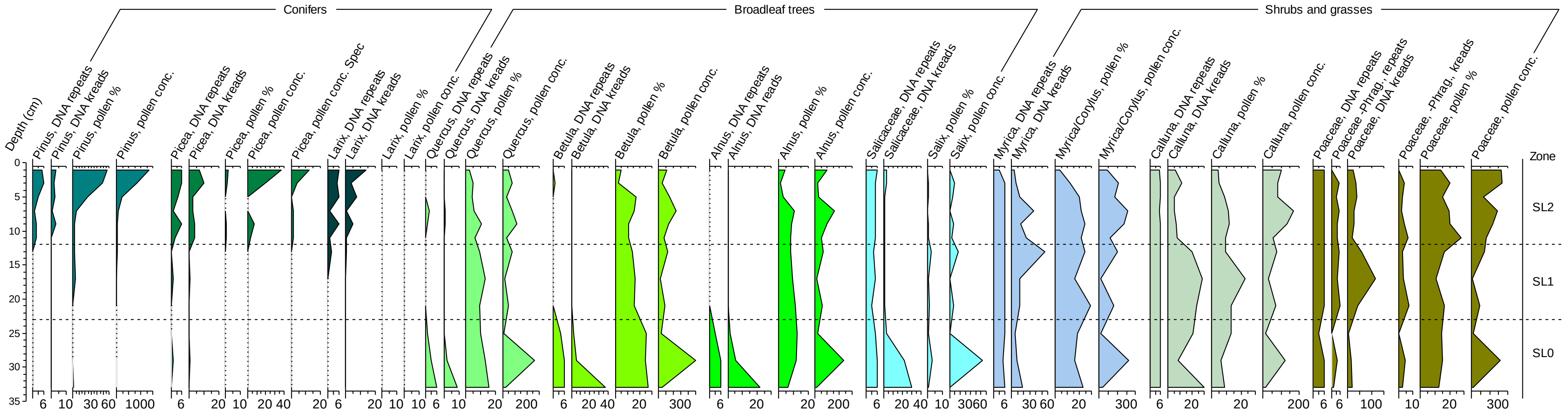

Fig. S5 Comparison of sedDNA repeats, sedDNA reads, pollen percentages and pollen concentrations from Spectacle Loch, Scotland. DNA reads are given in 1000 of reads (sum of all six PCR repeats). Pollen percentage values (pollen %) are based on total land pollen. Pollen concentrations values (pollen conc.) are given as 1000 of pollen per cm^3. ″Picea pollen conc. spec″ is based on a higher count of exotics than done for the other samples. ″Poaceae -Phrag.″ is the remaining Poaceae DNA repeats and reads when *Phragmites australis* is removed.

Table S1 Re-assignment of exotic taxa to native alternative provided by BLAST (100% similarity) or to higher taxonomic level when error in PCR is likely (differ only in subsequent number of identical bp). *Pedicularis parviflora* was removed as it had identical distribution among samples as the native *Pedicularis palustris*.

**Original assignment**	**Re-assignment**	**Comment**
Epilobium alsinifolium	*Epilobium obscurum*	BLASTed to native taxon
Lomelosia cretica	*Succisa pratensis*	BLASTed to taxon already present
Pedicularis parviflora	*(Pedicularis palustris)*	Identical distribution among samples
Pinguicula algida	*Pinguicula vulgaris*	BLASTed to taxon already present
Ranunculus ficariifolius	Ranuculaceae	PCR error
Ranunculus reptans	Ranuculaceae	PCR error
Vaccinium ovalifolium	*Vaccinium* sp.	PCR error

Table S2 SedDNA taxa recorded in both at least one sediment sample (out of 25 samples, 150 repeats) and one negative control sample (out of 12 samples, 72 repeats) and also in at least one of the sediment samples. Only records with ≥10 reads per repeat are included. Note that for common taxa the source of the DNA in the negative controls likely is the sediment samples themselves; in addition, the potential ecological impact of a false positive repeat is generally much smaller than for a rare taxon. Of the present taxa we consider the ones with a ratio of ≥0.35 between the relative occurrence in the negative controls in relation to the sediment samples as problematic, and these are removed from further interpretation. Most other taxa have very low values and/or high occurrence in the repeats, and the ecological interpretation for these is considered unproblematic.

**Taxa**	**Number of repeats in sediment samples**	**Fraction of repeats in sediments / fraction of repeats in negative controls**
Calluna vulgaris	140	0.01
Phragmites australis	140	0.01
Pinus	72	0.03
Molinia caerulea	90	0.05
Ranuculaceae	37	0.06
Nuphar lutea	149	0.07
Poeae	27	0.08
Carduinae	73	0.09
Pyreae	61	0.14
Alnus	15	0.14
Myriophyllum alterniflorum	135	0.14
Ranunculus	13	0.16
Potamogeton	140	0.18
Salicaceae	136	0.26
Nymphaeaceae	150	0.33
Musaceae	6	0.35
Holcus	6	0.35
Triticeae	11	0.38
Cucurbitaceae	5	0.42
Asteraceae	19	0.44
Veronica	6	0.69
Zea mays	15	0.83
Prunus	6	1.04
